# High Resolution Direction of Arrival (DOA) Estimation Based on Improved Orthogonal Matching Pursuit (OMP) Algorithm by Iterative Local Searching

**DOI:** 10.3390/s130911167

**Published:** 2013-08-22

**Authors:** Wenyi Wang, Renbiao Wu

**Affiliations:** Tianjin Key Lab for Advanced Signal Processing, Civil Aviation University of China, Tianjin 300300, China; E-Mail: rbwu@cauc.edu.cn

**Keywords:** direction of arrival estimation, compressive sensing, iterative local searching

## Abstract

DOA (Direction of Arrival) estimation is a major problem in array signal processing applications. Recently, compressive sensing algorithms, including convex relaxation algorithms and greedy algorithms, have been recognized as a kind of novel DOA estimation algorithm. However, the success of these algorithms is limited by the RIP (Restricted Isometry Property) condition or the mutual coherence of measurement matrix. In the DOA estimation problem, the columns of measurement matrix are steering vectors corresponding to different DOAs. Thus, it violates the mutual coherence condition. The situation gets worse when there are two sources from two adjacent DOAs. In this paper, an algorithm based on OMP (Orthogonal Matching Pursuit), called ILS-OMP (Iterative Local Searching-Orthogonal Matching Pursuit), is proposed to improve DOA resolution by Iterative Local Searching. Firstly, the conventional OMP algorithm is used to obtain initial estimated DOAs. Then, in each iteration, a local searching process for every estimated DOA is utilized to find a new DOA in a given DOA set to further decrease the residual. Additionally, the estimated DOAs are updated by substituting the initial DOA with the new one. The simulation results demonstrate the advantages of the proposed algorithm.

## 1. Introduction

The DOA (Direction of Arrival) estimation problem arises in many engineering applications, such as smart antennas, mobile communications, radio astronomy, sonar and navigation. There have been many high resolution DOA estimation algorithms; see [1] and the references therein. These algorithms can be broadly classified into three categories: data adaptive algorithms, subspace algorithms and maximum likelihood algorithms. The representative data adaptive algorithms are Capon [2] and APES (Amplitude and Phase Estimation) [3]. The subspace algorithms mainly include MUSIC (Multiple Signal Classification) [4] and ESPRIT (Estimation of Signal Parameters via a Rotational Invariance Technique) [5]. The deterministic maximum likelihood and stochastic maximum likelihood methods fall into maximum likelihood algorithms [6]. All these methods depend on some statistical properties of the data, e.g., a covariance matrix. There are also some algorithms without the need of estimating the covariance matrix. They utilize some learning techniques to obtain the statistical properties, e.g., Neural Networks (NN) [7] and Support Vector Machines (SVM) [8]. For these algorithms, a sufficiently large number of snapshots are needed to estimate or learn the statistical properties. In addition, for many algorithms, especially subspace algorithms, the strong correlation of the signals leads to serious performance degradation.

In recent years, compressive sensing algorithms have been recognized as a kind of novel high resolution DOA estimation algorithm. They are mainly based on the sparse property of the spatial spectrum when there is only a limited number of point sources [9]. More importantly, compressive sensing algorithms are still effective, even when there is only one snapshot, which is totally different from conventional high resolution DOA estimation algorithms. The existing references have demonstrated the advantages of this kind of algorithm from different viewpoints, e.g., convex optimization [9,10] and Bayesian statistics [11]. It is especially important in a high-dynamic environment. In that case, it is difficult, if not impossible, to obtain enough snapshots.

The representative compressive sensing algorithms include convex relaxation algorithms [12,13] and greedy algorithms [14,15], which have application in numerous fields [16]. It has been proven that the convex relaxation algorithms can accurately recovery a sparse signal under certain conditions. Compared with convex relaxation algorithms, greedy algorithms, such as OMP (Orthogonal Matching Pursuit) [14] and CoSaMP (Compressive Sampling Matching Pursuit) [15], are more computationally efficient, but suffer little performance degradation. Many of these algorithms can shown that the sparse signal also can be accurately recovered if the measurement matrix satisfies certain conditions. These conditions are, of course, more rigid than that of convex relaxation algorithms. In these greedy algorithms, the OMP algorithm is an early classic iterative method. In every iteration, it greedily chooses one support element based on the correlations between the residual and columns of the sensing matrix and, then, re-estimates the coefficients by utilizing the LS (Least Square) algorithm. Based on the same idea, many other greedy algorithms are proposed to decrease the complexity or increase the estimation precision.

As stated before, in order to ensure the success of convex relaxation algorithms and greedy algorithms, many works in the literature have already proven that the measurement matrix has to meet the RIP (Restricted Isometry Property) condition [17] or the incoherence condition [14]. These two conditions are closely related and can be derived from each other. This means that almost all algorithms will fail when the maximum pairwise coherence of the measurement matrix exceeds a constant. Unfortunately, in the DOA estimation problem, the columns of the measurement matrix are steering vectors according to different DOA. On the one hand, in order to obtain high resolution DOA estimation, the entire DOA space has to be divided into as many potential DOAs as possible, *i.e.*, as small a grid spacing as possible. However, on the other hand, the maximum pairwise coherence increases with decreasing of the grid spacing, even close to one. Thus, almost all algorithms will fail from too big of a maximum pairwise coherence. Therefore, it is interesting to propose a new compressive sensing algorithm to improve DOA estimation resolution.

In this paper, a new greedy algorithm in a compressive sensing framework is proposed to improve DOA estimation accuracy and resolution when there is only one snapshot. As with many other greedy algorithms, the proposed greedy algorithm is based on the OMP algorithm. The OMP algorithm is firstly used to obtain the initial estimated DOAs; then, an Iterative Local Searching process is utilized to improve DOA estimation accuracy. In each iteration, a local search is carried out for every DOA, *i.e.*, the support element. For every support element estimated by the OMP algorithm, a DOA set is defined, which includes some DOAs based on a given principle. Then, a local search in the DOA set is utilized to minimize the residual. The original DOA is replaced by the one corresponding to the minimal residual. The iterative process exists when all support elements do not change with local searching or the number of iteration exceeds a given constant. Once again, the signal is re-estimated based on the LS algorithm. Because every local searching process is limited in a small DOA set, the complexity of a new greedy algorithm will not be greatly improved. The simulation results demonstrate that the proposed algorithm can distinguish a DOA difference less than other convex relaxation algorithms and greedy algorithms. Specifically, without noise, the proposed algorithm can accurately recovery the two sources from adjacent DOAs, where the maximum pairwise coherent far surpasses the required coherence parameter. When there is noise, the proposed algorithm can still recovery two sources from the adjacent DOAs more accurately than other algorithms.

The reminder of the paper is organized as follows. The system model is presented in Section 2. The proposed algorithm is provided in Section 3. Section 4 demonstrates the performance and effectiveness of the provided algorithm. Section 5 concludes the paper.

## 2. System Model and Problem Formulation

Consider a uniform linear array (ULA) consisting of *M* identical and omnidirectional elements that are aligned and equally spaced on a line. Let the distance between two adjacent elements be one half of a wavelength. Assume that *K* far field narrow band sources is impinging upon the array from different angles. Then, the received signal, *y_m_* (*t*) by the *m*th (1 ≤ *m* ≤ *M*) array element is given as follows:

(1) $$y_{m}(t)=\sum_{k=1}^{K}a_{m}(\phi_{k}){s^{\prime }}_{k}\;(t)+n_{m}\;(t)$$

where *ϕ_k_* ∈ [−90°, 90°) (1 ≤ *k* ≤ *K*) is the impinging angle of source *k*, *s'_k_* (*t*) is the *k*th received signal at the reference element (e.g., the left-most element), *a_m_* (*ϕ_k_*) = e^−*j*2*π*(*m*−1)sin(*ϕ_k_*)^ is the steering vector element according to the DOA, *ϕ_k_*, for the array element, *m*, and *n_m_* (*t*) is complex Gaussian noise.

Let:

(2) $$\text{a}(\phi_{k})={[1,\text{e}}^{-j\pi \sin(\phi_{k})},\ldots ,{\text{e}}^{-j\pi (M-1)\sin(\phi_{k})]T}$$

(3) $${\text{A}}^{\prime }=[\text{a}(\phi_{1}),\ldots ,\text{a}(\phi_{K})]$$

(4) $${\text{s}}^{\prime }(t)={\left[{s^{\prime }}_{1}(t),\ldots ,{s^{\prime }}_{K}(t)\right]}^{T}$$

(5) $$\text{y}(t)={\left[y_{1}(t),\ldots ,y_{M}\;(t)\right]}^{T}$$

and:

(6) $$\text{n}(t)={\left[n_{1}(t),\ldots ,n_{M}\;(t)\right]}^{T}$$

where (*)*^T^* denotes the matrix transpose.

Then, the received signal of the array can be denoted in a matrix formulation as follows:

(7) $$\text{y}\;(t)={\text{A}}^{\prime }{\text{s}}^{\prime }(t)+\text{n}\;(t)$$

where A′ is often called the steering matrix.

The snapshot vector can be obtained by sampling the received signal at discrete times:

(8) $$\text{y}={\text{A}}^{\prime }{\text{s}}^{\prime }+\text{n}$$

where the notation in time dependence is omitted, as we focus on only one snapshot case.

The DOA estimation problem is to find the DOA, *ϕ_k_*, by utilizing the received signal, y. As stated before, many high resolution DOA estimation algorithms depend on some statistical properties, e.g., the covariance matrix. Thus, a sufficiently large number of snapshots are necessary to accurately obtain those statistical properties. However, in some cases, it is difficult to obtain enough snapshots, e.g., a high-dynamic target. Thus, almost all high resolution DOA estimation algorithms will fail when there are few snapshots. Fortunately, compressive sensing algorithms can be used to estimate DOA, even with only one snapshot.

Compressive sensing considers the problem of estimating the signal based on the measurements of the form, **z** = **Hx** + **e**, where **H** is a measurement matrix and e is a noise vector. Specifically, for the measurement matrix, the number of rows is far less than the number of columns. If **x** is sparse or compressible and **H** satisfies certain conditions, then compressive sensing algorithms provide a mechanism to recover the signal, x, from the measurement vector, z, efficiently and robustly. The measurement matrix has to satisfy either RIP or the incoherence condition, which can be derived from each other. Here, we focus on the incoherence condition.

One commonly used characterization of incoherence in the compressive sensing framework is in terms of the mutual coherence, *μ*. Let the pairwise coherence between the *k*th and *l*th column be:

(9) $$\mu (k,l)=\frac{\left|\langle {\text{H}}_{.k},{\text{H}}_{.l}\rangle \right|}{\left|{\text{H}}_{.k}\right|\left|{\text{H}}_{.l}\right|}$$

where **H***_.k_* denotes the *k*th column of measurement matrix **H**.

The mutual coherence of **H** is the maximum pairwise coherence among all pairs of columns:

(10) $$\mu (\text{H})=\underset{k\neq l}{\max}\mu (k,l)$$

In order to utilize compressive sensing algorithms to solve the DOA estimation problem, the received signal model has to be transformed into a sparse representation, which fits the compressive sensing model. The key idea is to divide the total DOA space into a potential DOA set with a given grid spacing, e.g., Θ = {−90°,−89°, ⋯, 88°, 89°}, with grid spacing 1°. The grid spacing, 1°, is used in the following parts, if not stated otherwise. Thus, the steering matrix, **A**′, can be transformed to an overcomplete dictionary, **A**, where the number of columns is equal to the size of the potential DOA set, Θ, e.g., *N* = 180. As with many other references, it is assumed that the DOAs of sources are included in the potential DOA set, e.g., *θ_k_* ⊆ Θ for all *k*. If the potential DOA set does not include the DOAs of sources, an effective way is to gradually decrease the grid spacing to enlarge the size of the potential DOA set [9].

Then, the upper equation can be reformulated as:

(11) y=As+n

The vector, s, denotes a new vector, which relates to the signal vector, s′, as follows:

(12) $${\text{s}}_{n}=\{\begin{array}{ll} {s^{\prime }}_{k}, & \Theta_{n}=\phi_{k}\\ 0, & \text{otherwise} \end{array}$$

where *s_n_*(1 ≤ *n* ≤ *N*) and Θ*_n_* are the *n*th element of the sparse vector, s, and the potential DOA set, Θ, respectively.

As the number of sources is far less than the size of the potential DOA set (*N* ≫ *K*), most of s are zero. Thus, s is a sparse vector. Then, the DOA estimation problem has been transformed as a standard compressive sensing problem, where A and s are the measurement matrix and the sparse vector, respectively. Therefore, all compressive sensing algorithms can be utilized to obtain DOA estimation. However, as the special structure of the measurement matrix, it will bring a new problem, which will be discussed in detail in the next section.



**Algorithm 1:** The OMP (Orthogonal Matching Pursuit) algorithm.
** Input:** y, A, *K*;** Initial state:** r^0^ = y, Π^0^ = ∅, *l* = 0;** Repeat**   *l* = *l* + 1;  match step:   b*^l^* = A^H^r*^l^*^−1^;  identify step:   Π*^l^* = Π*^l-1^* ∪ {arg max*_j_* |b*^l^*(*j*)|_2_};  update step:   *s^l^* = arg min_**z**:supp (**z**)⊆Π^*l*^_ ║y − **Az**║_2_;   **r***^l^* = **y** − **As***^l^*;** Until** *l* = *K*;** Output:** *s^K^*, Π*^K^*.


## 3. Improved OMP Algorithm by Iterative Local Searching

### 3.1. OMP Algorithm and Discussion

As the DOA estimation problem has been transformed as a compressive sensing problem, the existing convex relaxation algorithms and greedy algorithms can be utilized to solve it. The representative convex relaxation algorithm is the BPDN (Basis Pursuit Denoising) algorithm [13], which estimates the DOAs by the following optimization problem:

(13) $$\begin{array}{ll} \min_{\text{s}} & {\left\|\text{s}\right\|}_{1} \end{array}$$

(14) $${\begin{array}{cc} \text{subject to} & \left\|\text{y}-A\text{s}\right\| \end{array}}_{2}\leq \varepsilon$$

where ║s║_1_ is the *l*_1_ norm of vector s: ║s║_1_ = Σ*_i_*|s*_i_*| and *ε* is a given parameter related to the noise vector, n. When there is no noise, *ε* is set to zero. Once s is estimated, the DOAs of sources can be obtained based on the corresponding locations of peaks.

Another kind of compressive sensing algorithm is greedy algorithms. Compared with convex relaxation algorithms, greedy algorithms have obvious advantages in complexity. In all greedy algorithms, the most typical one is the OMP algorithm, where the main idea is to obtain a support element, in each iteration, by finding the maximum correlation between the residual and columns of the measurement matrix. As with many other greedy algorithms, the proposed algorithm is also based on the OMP algorithm. For the integrity of this paper, the DOA estimation based on the OMP algorithm is stated in a table called “Algorithm 1: The OMP (Orthogonal Matching Pursuit) algorithm”. Here, (*)*^H^* denotes the matrix Hermitian transpose. It is also noted that the sparse vector, s, is estimated by the OMP algorithm, and the estimated DOAs are the corresponding ones of the *K* nonzero entities.

As stated before, the success of convex relaxation algorithms and greedy algorithms is limited by the mutual coherence of the measurement matrix. Here, it is apparent that the mutual coherence is greater than the desired limitation. Though there are some references that proposed several improved algorithms to account for this problem [18], they still require enough DOA difference between two sources.

However, in the ideal case, it is noted that the DOAs can be accurately recovered by the following *l*_0_-norm optimization problem if **A** is a full column rank and the number of columns satisfies *N* ≥ 2*K*.

(15) $$\begin{array}{ll} \min_{\text{s}} & {\left\|\text{s}\right\|}_{0} \end{array}$$

(16) $$\begin{array}{cc} \text{subject to} & \text{y}=\text{A}s \end{array}$$

where ║s║_0_ denotes the number of the nonzero entries of s. At the same time, it is easy to prove that **A** is a Vandermonde matrix. Therefore, the matrix, **A**, is a full column rank, and the *l*_0_-norm optimization problem can accurately recover the DOAs in the ideal case. Though the *l*_0_-norm optimization problem is an NP-hard problem, it tells us that there is still space to improve the DOA estimation accuracy, which motivates the proposed algorithm.

### 3.2. The Proposed Algorithm

The main idea of the DOA estimation based on the OMP algorithm is to obtain a DOA, in each iteration, by finding the maximum correlation between the residual and steering vectors according to different DOAs, which are not included in the estimated DOA set. The sparse vector will be re-estimated based on the updated DOAs by utilizing the LS algorithm. The residual will also be recomputed by subtracting the contributions of the estimated sources from the updated DOAs. The total number of iterations is the number of sources, e.g., *K*. As stated before, theoretical results show that the mutual coherence of the measurement matrix limits the success of the OMP algorithm. When there are two sources from two adjacent DOAs, the situation becomes worse. Then, the OMP algorithm cannot distinguish between the two sources. Here, we will try to find the reason, from the point of view of array signal processing, by going deep into each iteration of the OMP algorithm.

In the array signal processing community, it is well known that the classic DAS (Delay-And-Summation) algorithm estimates the DOAs by searching the peaks of all correlations between the received signal and all possible steering vectors according to different DOAs. Therefore, in each iteration, the OMP algorithm is very similar to the conventional DAS algorithm. The difference is that the OMP algorithm only finds the maximum peak in each iteration, and the DAS algorithm finds all peaks at once. There have been many works in the literature about DOA resolution of the DAS Algorithm [1], which show that the DOA resolution of the DAS algorithm is the Rayleigh limit. This means that the DAS algorithm cannot distinguish between two sources, where the DOA difference is less than Rayleigh limit. In this case, as the strong correlation between the steering vectors of the two DOAs, the DAS algorithm will find one peak locating between the two real DOAs. The two sources are recognized as only one source and cannot be distinguished. Based on the same reason, in some iterations, the OMP algorithm will find one peak locating between the two DOAs. The DOA corresponding to the peak will be recognized as an estimated DOA. After that, the contribution of the estimated signal will be subtracted from the residual. Once again, as the strong correlation between the steering vectors of the two DOAs and the estimated DOA, the two real DOAs are impossible to be found in other iterations.

Though some improved algorithms have been proposed based on the OMP algorithm, many references show that almost all algorithms are still limited by the mutual coherence of the measurement matrix. One typical improved algorithm is the CoSaMP algorithm [15], where the number of iterations is decreased by finding more than one DOA in one iteration. However, the CoSaMP algorithm has a more rigid mutual coherence requirement, which leads to a lower DOA resolution. Therefore, the CoSaMP algorithm still cannot distinguish between two sources, where the DOA difference is far less than the Rayleigh limit.

However, the theoretical result of the *l*_0_-norm optimization problem tells us that the DOAs with small difference still can be distinguished, at least for the ideal case. Through searching all possible DOA groups, the DOAs can be accurately recovered, even when the DOA difference is far less than the Rayleigh limit. However, the complexity of exhaustive searching is exponential in *N*, and it has been proven as an NP-hard problem. This motivates us to propose a greedy algorithm to improve the DOA resolution by local searching.

As with many DOA estimation papers [6], the number of sources is assumed to be known. The assumption is rational when the source number is known *a priori*, e.g., target tracking. Thus, the estimation of the source number is not necessary, and the proposed algorithm is still effective. If this is not true, there are still some references about the estimation of the source number [19] that may be utilized to tackle this problem. We hope to continue this study in another paper. Firstly, the OMP algorithm is used to obtain the initial DOA estimation. Then, Iterative Local Searching is utilized to further improve the DOA estimation performance by an outer iteration and an inner iteration. In each outer iteration, all estimated DOAs are optimized once. In the inner iteration, for every estimated DOA, a local search is sequentially taken to find a DOA in the given searching range to minimize the residual. In a local search, other estimated DOAs hold fixed, except the current DOA. Then, the initial DOA is replaced with the new one, according to the minimal residual. The iteration process exists when the estimated DOAs do not change or the number of iterations is greater than the maximum iteration number. After that, the sparse vector will be re-estimated based on the updated DOAs. From the point of view of optimization theory, the proposed algorithm can be recognized as an iterative local optimizing algorithm, where the initial values are given by the OMP algorithm. In each iteration, all variables are sequentially optimized to find a local optimal solution. Here, the variables are the support elements of the sparse vector, s. Thus, the complexity is decreased. When the estimated DOAs do not change again, the local optimization process converges to a local optimal solution. For the qth inner iteration in the *p*th outer iteration, the local searching process can be expressed as follows:

(17) $$\begin{array}{cc} q^{\prime }=\arg\underset{q^{\prime }:\sup\text{p}(\text{z})=\wedge^{p-1}\backslash \{\wedge_{q}^{p-1}\}\cup \{q^{\prime }\}}{\min}{\left\|\text{y}-\text{Az}\right\|}_{2}, & q^{\prime }\in E_{\wedge_{q}^{p-1}} \end{array}$$

where supp (**z**) is the support set of **z**, Λ*^p^*^−1^ is the support set estimated in the (*p*− 1)th outer iteration and $\wedge_{q}^{p-1}$ is the corresponding *q*th element; $E_{\wedge_{q}^{p-1}}$ is a given DOA set for the support $\wedge_{q}^{p-1}$, *i.e.*, the searching range. As with other algorithms, once the sparse vector is estimated, the DOAs of the sources can be obtained as the DOAs corresponding to the nonzero elements of the sparse vector. The details can be referred to in the table called “Algorithm 2: The ILS-OMP (Iterative Local Searching - Orthogonal Matching Pursuit) algorithm”.

So far, the remaining problem to be solved is how to set the searching range, *i.e.*, $E_{\wedge_{q}^{p-1}}$. Once again, this is based on the analysis of the DAS algorithm. As stated before, when there are two sources from adjacent DOAs, the OMP algorithm will find a DOA located between the two reals DOAs as an estimated DOA. It is also worth reminding that the DOA estimation resolution of the OMP algorithm, as with the DAS algorithm, is the Rayleigh limit. Thus, the real DOAs are around the estimated DOA, and the range is the Rayleigh limit. Based on this conclusion, the searching range is set as the Rayleigh limit, whose center is the estimated DOA. The Rayleigh limit is related to the number of array elements. Thus, the searching range is set as follows:

(18) $$E_{\wedge_{q}^{p-1}}=\left\{\left\lfloor \wedge_{q}^{p-1}-\frac{\Delta }{2}\right\rfloor ,\ldots ,\wedge_{q}^{p-1}-1,\wedge_{q}^{p-1},\wedge_{q}^{p-1}+1,\ldots \left\lceil \wedge_{q}^{p-1}+\frac{\Delta }{2}\right\rceil \right\}$$

where ∆ is the Rayleigh limit and ⌊*⌋ and ⌈*⌉ are the floor function and ceiling function, respectively. Apparently, the searching range decreases with increasing the number of array elements.

According to the complexity, as the local searching is limited in a small searching range, the complexity will not be greatly increased by the local searching process. At the same time, in most cases, the simulations show that the support elements will not change after only several iterations. Therefore, the complexity of the proposed algorithm is slightly higher than the OMP algorithm.



**Algorithm 2:** The ILS-OMP (Iterative Local Searching - Orthogonal Matching Pursuit) algorithm.
**  Input:** y, **A**, *K*, *MaxIter Num*;**  Conventional OMP algorithm:****    Initial state:** r^0^ = y, Π^0^ = ∅, *l* = 0;**    Repeat**     *l* = *l* + 1;    match step:     **h***^l^* = **A**^H^**r***^l^*^−1^;    identify step:     Π*^l^* = Π*^l^*^−1^ ∪ {arg max*_j_* |**h***^l^*(*j*)|_2_};    update step:     *s^l^* = arg min_**z**:supp(**z**)⊆Π^*l*^ ║**y** − **Az**║_2__;     **r***^l^* = **y** − **As***^l^*;  **Until**   *l* = *K*;  **Output:**  *s^K^*, Π*^K^*. **Iterative Local Searching:**  **Initial state:**  Λ^0^ = Π*^K^*, *p* = 0, *q* = 0;  **Outer Iteration:****  Repeat**   *p* = *p* + 1;   V*^p^* = Λ*^p^*^−1^;  **Inner Iteration:**   *q* = 0;  **Repeat**   *q* = *q* + 1;   Searching step:    
$\begin{array}{cc} q^{\prime }=\arg\min_{q^{\prime }:\sup\text{p}(\text{z})=\wedge^{p-1}\backslash \{\wedge_{q}^{p-1}\}\cup \{q^{\prime }\}}\;{\left\|\text{y}-\text{Az}\right\|}_{2}, & q^{\prime }\in E_{\wedge_{q}^{p-1}} \end{array};$  
$\wedge^{p-1}=\wedge^{p-1}\backslash \{\wedge_{q}^{p-1}\}\cup \{q^{\prime }\};$**  Until**  *q* = *K*;  Λ*^p^* = Λ*^p^*^−1^;** Until**  Λ*^p^* = V*^p^* or *p* ≥ *MaxIter Num*; ŝ = arg min_**z**:supp(**z**)=Λ*^p^*_ ║**y** − **Az**║_2_;** Output:** ŝ, Λ*^p^*.


As an extreme case, the proposed algorithm estimates the DOAs by utilizing only one snapshot. However, when there is more than one snapshot, the proposed algorithm is still effective, where the OMP algorithm will be replaced with the OMP algorithm for multiple measurement vectors [20]. The performance of the algorithm will be improved with increasing of the number of snapshots [20]. On the other hand, a uniform linear array is adopted in this paper, where a one-dimensional local search is included in each iteration. For the planar array, in addition to the OMP algorithm, which has to be utilized to estimate the azimuth and elevation DOA, a two-dimensional local search (azimuth and elevation) in one iteration is necessary to estimate the azimuth and elevation DOA.

## 4. Simulation Results

In this section, several numerical simulation results are presented to illustrate the performance of the proposed algorithm. Unless stated otherwise, a uniform linear array is assumed, and the number of array elements is *M* = 12. The array element spacing is one half of the wavelength. The Rayleigh limit is approximately set as ∆ = (100/*M*)°. All sources are modeled as an identical constant-modulus signals, and the additive noise at all array elements is modeled as complex white Gaussian noise with identical variance. The *SNR* (signal-to-noise ratio) is defined as *SNR* = *P*/*σ*^2^, where *P* is the power of one source and *σ*^2^ is the covariance of the noise.

As only one snapshot is assumed, many conventional DOA estimation algorithms based on some statistical properties are not applicable. Therefore, the proposed algorithm is only compared with one typical greedy algorithm and one typical convex relaxation algorithm, e.g., the OMP algorithm and the BPDN algorithm. For the proposed algorithm, the maximum number of iterations is set as 10, e.g., *MaxIter Num* = 10. On the other hand, in order to remove the impact of noise, the proposed algorithm is evaluated in two cases (without and with noise).

**Figure 1.**
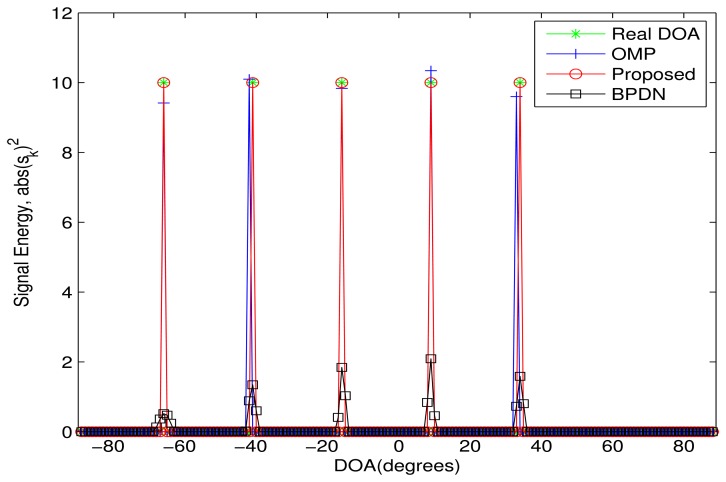
The performance comparison in the ideal case. Five sources are assumed from the Directions of Arrival (DOAs), {−66^◦^, −41^◦^, −16^◦^,9^◦^, 34^◦^}. The power of the sources and noise is 10 *dB* and zero, respectively. The number of array elements is *M* = 12, and the number of snapshots is one.

In the first simulation, the proposed algorithm is evaluated in an ideal case, where five sources are included. The power of sources and noise is set as 10 *dB* and zero, respectively. Here, the DOA difference between all sources is assumed greater than the Rayleigh limit. Five DOAs of sources are {−66°,−41°,−16°, 9°, 34°}. The grid spacing is 1° and *μ*(**A**) = 0.999998635882. The simulation result is shown in Figure 1. In the simulation, the proposed algorithm will converge only after three iterations. It is clear that all three algorithms can obtain the DOAs. Specifically, the proposed algorithm perfectly estimates all DOAs. However, for the OMP algorithm, there is still a small DOA estimation error, especially for the second and fifth sources. This means that the OMP algorithm cannot perfectly estimate the DOAs, even in the ideal case. Though the BPDN algorithm also can accurately estimate all DOAs, the estimated sparse vector of the proposed algorithm is sparser than that of the BPDN algorithm.

**Figure 2.**
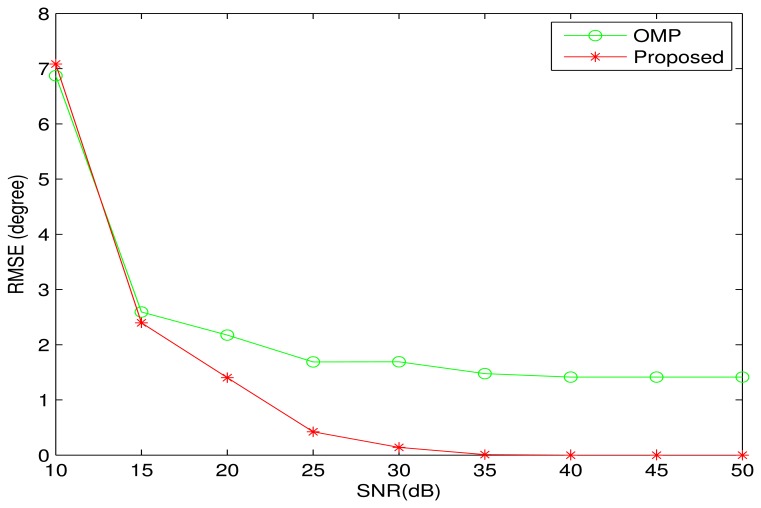
Root mean square error as a function of the signal-to-noise ratio (*SNR*). Five sources are assumed from the DOAs: {−66^◦^, −41^◦^, −16^◦^, −9^◦^, −34^◦^}. The number of array elements is *M* = 12, and the number of snapshots is one. The curves were obtained by averaging the results of 100 independent simulation runs.

In the noise case, the proposed algorithm is compared with the OMP algorithm in terms of RMSE (Root Mean Square Error). Here, all DOAs are set to be the same as that in Figure 1. In other simulations, where there are two sources from the adjacent DOA, the BPDN algorithm cannot distinguish between the two sources, and the DOA error cannot be evaluated. Therefore, the BPDN algorithm will not be included in all noise cases. Figure 2 shows the simulation result with 100 Monte-Carlo trials. It is clear that the proposed algorithm performs better than the OMP algorithm. When *SNR* > 35 *dB*, the proposed algorithm can perfectly recover all DOAs. However, the DOA estimation error of the OMP algorithm tends to a nonzero value with increasing of *SNR*, which is consistent with the simulation result in Figure 1.

**Figure 3.**
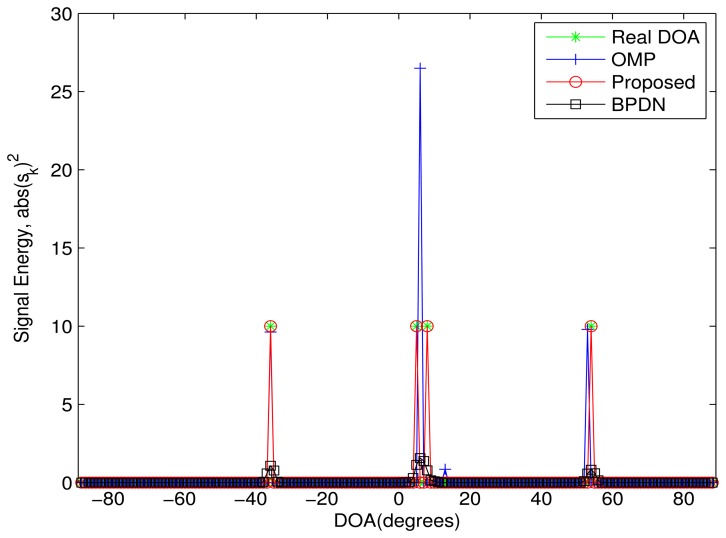
The performance comparison in the ideal case. Four sources are assumed from the DOAs: {−36^◦^, 4^◦^, 8^◦^, 54^◦^}. The power of the sources and noise is 10*dB* and zero, respectively. The minimal DOA difference is 4^◦^.

In order to evaluate the DOA resolution of the proposed algorithm, Figure 3 shows the simulation result in an ideal case, where there are two sources from the adjacent DOA. The DOAs of three sources are {−36^◦^, 4^◦^, 8^◦^, 54^◦^}, where the minimal DOA difference (e.g., 4^◦^) is far less than the Rayleigh limit. The power of the sources and noise are set as 10 *dB* and zero, respectively. The proposed algorithm perfectly estimates all DOAs again. The BPDN algorithm cannot distinguish between the two sources from the adjacent DOA. For the OMP algorithm, a DOA between the two real DOAs is recognized as an estimated DOA, which leads to an improved error.

**Figure 4.**
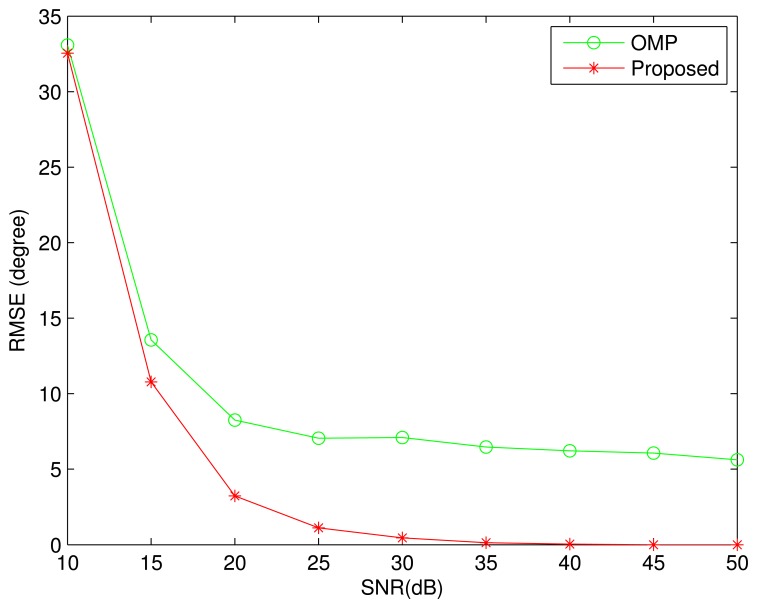
Root mean square error as a function of *SNR*. Four sources are assumed from the DOAs: {−36^◦^,4^◦^,8^◦^, 54^◦^}. The minimal DOA difference is 4^◦^. The curves were obtained by averaging the results of 100 independent simulation runs.

The same conditions are adopted in the next simulation, except the *SNR*. Figure 4 shows the simulation result with 100 Monte-Carlo trials. As with that in Figure 2, the proposed algorithm performs better than the OMP algorithm. When *SNR* > 35 *dB*, the proposed algorithm almost can perfectly recover all DOAs. For the OMP algorithm, the DOA estimation error decreases slowly with increasing of the *SNR*.

In the following two simulations, the grid spacing is reduced to 0.1°, which is far less than that of all the above simulations. Thus, the mutual coherence is *μ* (**A**) = 0.999999999864, and the mutual coherence of the measurement matrix is also greater than that of other simulations. As before, the proposed algorithm is evaluated in two cases, respectively. The DOAs of sources are assumed to be {−36.1°, 4.1°, 8.1°, 54.1°}, where the minimal DOA difference is the same as that in Figures 3 and 4, e.g., 4°.

**Figure 5.**
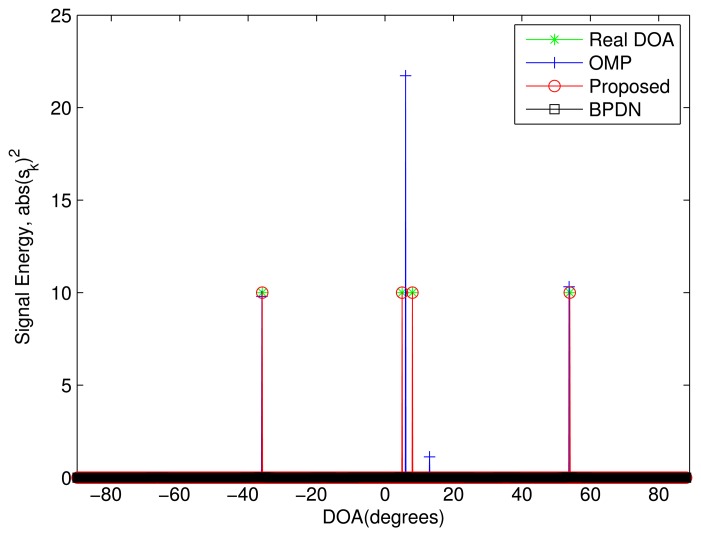
The performance comparison in the ideal case. Four sources are assumed from the DOAs: {−36.1^◦^, 4.1^◦^, 8.1^◦^, 54.1^◦^}. The power of the sources and noise is 10 *dB* and zero, respectively. The minimal DOA difference is 4^◦^, and the grid spacing is 0.1^◦^.

**Figure 6.**
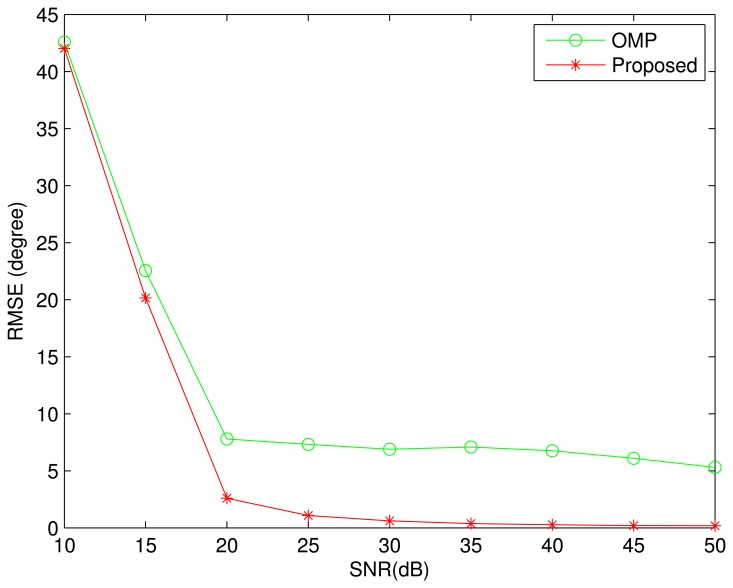
Root mean square error as a function of *SNR*. Four sources are assumed from the DOAs: {−36.1^◦^, 4.1^◦^, 8.1^◦^, 54.1^◦^}. The minimal DOA difference is 4^◦^, and the grid spacing is 0.1^◦^. The curves were obtained by averaging the results of 100 independent simulation runs.

Figure 5 shows that the proposed algorithm still can accurately recover the DOAs in the ideal case. Other algorithms cannot distinguish between the two sources from the adjacent DOAs. It should be noted that the estimated signal energy is not all zeros. As with the large mutual coherence, the estimated signal is not sparse enough, and the nonzero elements are very close to zero, but not equal to zero. The simulation in the noise case is shown in Figure 6. It is clear that the proposed algorithm performs better than the OMP algorithm. Different from the simulation in Figure 4, the proposed algorithm cannot perfectly recover all DOAs, even when *SNR* > 35 *dB*, which indicates that the performance of the proposed algorithm degrades with decreasing of the grid spacing. However, the DOA errors are still far less than those of the OMP algorithm, which demonstrates the advantages of the proposed algorithm.

**Figure 7.**
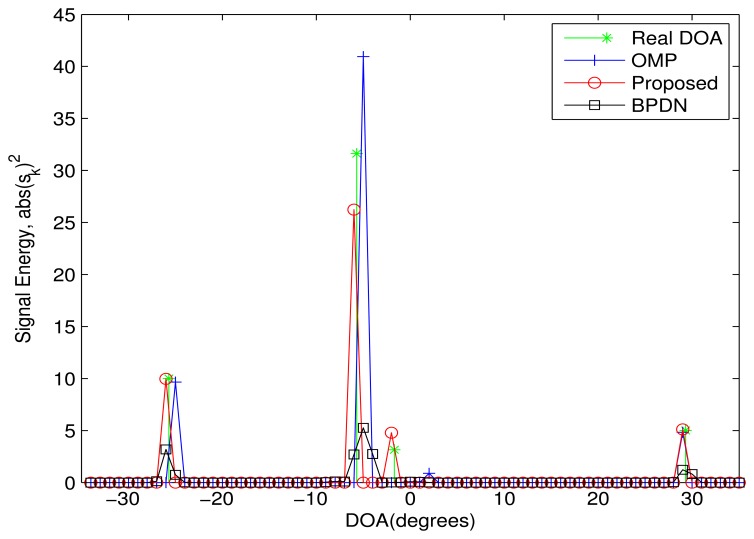
The performance comparison in the ideal case. Four sources are assumed from the DOAs {−25.7^◦^, −5.7^◦^, −1.7^◦^, 29.3^◦^}. Four sources have different power, as 10 *dB*, 15 *dB*, 5 *dB* and 7 *dB*, respectively. The minimal DOA difference is 4^◦^, and the grid spacing is 1^◦^. The number of array elements is *M* = 12, and the number of snapshots is one.

All previous simulations assume that all DOAs are exactly located on the DOA grids. However, as with many other algorithms, the performance of the proposed algorithm will degrade if it is not true [21]. Figure 7 compares the proposed algorithm with the OMP algorithm and the BPDN algorithm in an ideal case. The four DOAs are assumed as {−25.7^◦^, −5.7^◦^, −1.7^◦^, 29.3^◦^}, and the grid spacing is 1^◦^. Thus, all DOAs are not exactly located on the grids. Additionally, the four sources are assumed to have different power, as 10 *dB*, 15 *dB*, 5 *dB* and 7 *dB*, respectively. The simulation result shows that the proposed algorithm still performs better than the other two algorithms.

**Figure 8.**
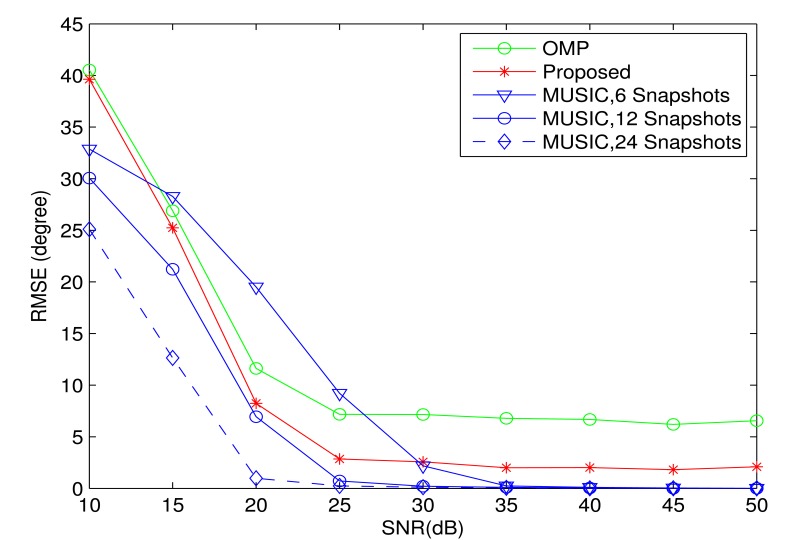
Root mean square error as a function of *SNR*. Four sources are assumed from the DOAs: {−25.7^◦^, −5.7^◦^, −1.7^◦^, 29.3^◦^}. The minimal DOA difference is 4^◦^, and the grid spacing is 1^◦^. The number of array elements is *M* = 12. The curves were obtained by averaging the results of 100 independent simulation runs.

Figure 8 compares the proposed algorithm with the other two algorithms in terms of RMSE. Here, the DOAs and grid spacing are assumed to be the same as that in Figure 7. The four sources have equal power. At the same time, the MUSIC algorithm is also included as a conventional DOA estimation algorithm. However, as stated before, there is not enough snapshots in the high dynamic environment. In order to compare the performance with the conventional high resolution DOA estimation algorithm, the MUSIC algorithm is included with different snapshots. It is noted that the proposed algorithm and the OMP algorithm are still based on only one snapshot. It is clear that the proposed algorithm still performs better than the OMP algorithm and the MUSIC algorithm with six snapshots under some SNR conditions. When the number of snapshots is larger than 12, the performance of the MUSIC algorithm is always better than the proposed algorithm. However, it is difficult, if not impossible, to obtain so many snapshots in the high dynamic environment.

## 5. Conclusions

This paper proposes a new DOA estimation algorithm to improve DOA resolution based on a classical greedy compressive sensing algorithm, e.g., the OMP algorithm. Firstly, the DOA estimation problem is transformed as a standard compressive sensing problem. As with the strong coherence between the steering vectors, the success of compressive sensing algorithms, including convex relaxation algorithms and greedy algorithms, is limited by the mutual coherence of the measurement matrix. However, the theoretical result of the *l*_0_-norm optimization problem tells us that the DOAs can be accurately estimated, at least in the ideal case. Then, an OMP-based algorithm is proposed to improve high resolution by Iterative Local Searching. The initial values are obtained by the OMP algorithm. In each iteration, a local search for every estimated DOA is utilized to improve estimation performance. The simulation results show that the proposed algorithm has better performance than other algorithms. Specifically, in the ideal case, the proposed algorithm can accurately estimate the DOAs, even if their DOA difference is far less than the Rayleigh limit. When there is noise, the proposed algorithm still performs better than other algorithms.
